# Enzymatic Hydrolysis and Simultaneous Extraction for Preparation of Genipin from Bark of *Eucommia ulmoides* after Ultrasound, Microwave Pretreatment

**DOI:** 10.3390/molecules201018717

**Published:** 2015-10-15

**Authors:** Lili Li, Yupin Guo, Lianfei Zhao, Yuangang Zu, Huiyan Gu, Lei Yang

**Affiliations:** 1 Key Laboratory of Forest Plant Ecology, Ministry of Education, Northeast Forestry University, Harbin 150040, China; E-Mails: lilili001@126.com (L.L.); zhaolianfei2013@126.com (L.Z.); zygorl@126.com (Y.Z.); 2 State Engineering Laboratory for Bioresource Eco-Utilization, Northeast Forestry University, Harbin 150040, China; 3 College of Animal Science and Technology, Hebei North University, Zhangjiakou 075000, China; E-Mail: yguohb@163.com; 4 School of Forestry, Northeast Forestry University, Harbin 150040, China

**Keywords:** enzymatic hydrolysis and simultaneous extraction, *Eucommia ulmoides* bark, genipin, ultrasound/microwave irradiation pretreatment

## Abstract

A continuous process based on the combination of ultrasounds and/or microwaves pretreatments followed by enzymatic hydrolysis and simultaneous extraction (EHSE) has been proposed to recover genipin from *Eucommia ulmoides* bark. At first, in the pretreatment step, the mixture of 1.0 g dried bark powder and 10 mL deionized water were irradiated by microwave under 500 W for 10 min. Then, in hydrolysis step, the optimal conditions were as follows: 0.5 mg/mL of cellulase concentration, 4.0 pH of enzyme solution, 24 h of incubation time and 40 °C of incubation temperature. After incubation, 10 mL ethanol was added to extract genipin for 30 min by ultrasound. After EHSE treatment, the yield of genipin could reach 1.71 μmol/g. Moreover, scanning electron micrographs illustrated that severe structural disruption of plant was obtained by EHSE. The results indicated that the EHSE method provided a good alternative for the preparation of genipin from *Eucommia ulmoides* bark as well as other herbs.

## 1. Introduction

*Eucommia ulmoides* (*E. ulmiodes*), a unique kind of precious tree in China, is widely cultivated in the southwest and the Yangtze River basin of China. *E. ulmoides* is also occasionally planted in botanical gardens in Europe, North America and elsewhere. It shows various pharmacological properties, including anti-oxidative [1], protection against cytotoxicity [2], benefits the liver and kidney [3], lowers blood hypertension [4], hypoglycemic [5], hypnotic and anticonvulsant activities [6], neuroprotective effects [7], weight loss [8], strengthening tendons and bones [9], and other pharmacological effects [10]. The bark of *E. ulmoides* is one of the oldest tonic traditional Chinese medicines and officially listed in the Chinese Pharmacopoeia. Genipin, an important component in *E. ulmoides* bark, is used as blue pigment in the food industry [11]. Additionally, genipin is an excellent naturally occurring, low-cytotoxic, higher biocompatibility [12] and biodegradable cross-linking agent, which can combine with protein, collagen [13], gelatin [14] and chitosan [15] to produce biological materials such as wound-dressing [16], artificial bone [17] and extracellular matrices [18]. Furthermore, genipin has been reported to possess many pharmacological actions such as anti-inflammatory [19], inhibiting hepatocyte apoptosis and uncoupling protein 2 and protecting hippocampal neurons from Alzheimer’s amyloid β-protein toxicity [20]. Geniposide, another important substance of *E. ulmoides* bark, can undergo hydrolysis to form genipin by β-glucosidase [21,22].

For preparing genipin, traditional methods can often be divided into the following steps: (1) geniposide in the bark of *E. ulmoides* is extracted by extraction solution of ethanol; (2) the extraction solution is separated from bark of *E. ulmoides*; (3) the extracting solution is distilled to remove ethanol and obtain extractum; (4) the extractum is redissolved in deionized water and mixed with β-glucosidase solution; and (5) the mixture is incubated at appropriate conditions for hydrolysis of geniposide to form genipin. For above-mentioned method, the yield of genipin is low due to poor permeability of plant materials and complicated separation process.

Plant cell walls have dense structures, composed of cellulose, hemicellulose, pectin and other substances. Ultrasound, microwave and enzymatic hydrolysis methods have been applied in order to destroy the cell wall structure, resulting in partial collapse and expansion that reduces the mass transfer barrier between the extraction solvent and the plant cell wall, which accelerate the dissolution rate of the active ingredients, and thus improve the extraction efficiency and shorten the extraction time. Among these, ultrasound irradiation method has been used to obtain valuable compounds, such as polyphenols, proteins, anthocyanins, coloring pigments, glucosinolates and isothiocyanates [23,24,25,26,27,28], due to its advantages including greater penetration, shorter treatment time and higher product yields. The cavitation effects of ultrasound can lead to the physical effects of velocity/pressure shockwaves, in which the disruption of the cell wall occurred to improve the release of target compounds [29,30]. Some applications of microwaves were reported to enhance the yields of polyphenols, pigments, lipid, and oil [29,30,31]. Microwave energy that is applied to heat the material inside the cell and water vapor can generate higher pressure in the bubbles, and thus destroy the cell wall and facilitate the release of intracellular compounds into the solvent. Microwave treatment is characterized as being rapid, low energy, and efficient [30]. Although microwave and ultrasound methods have been used widely to treat plant samples, the outcomes are generally not ideal, often requiring multiple extractions.

The aim of the work in this paper was to develop an efficient ultrasound or microwave irradiation pretreatment for improvement of the permeability of cell walls, followed by enzymatic hydrolysis and simultaneous extraction (EHSE) process for preparing genipin from bark of *E. ulmoides*. Firstly, the plant material was mixed with water and pretreated with microwave, ultrasound or their combination irradiation to enhance permeability, promoting penetration of enzyme. Secondly, enzyme was added into the mixture to further decompose cell wall to reduce the mass transfer barrier of extraction solvent, while, at the same time, geniposide was hydrolyzed into genipin. Thirdly, ethanol was added to extract genipin. Finally, the extraction solution containing genipin was separated from the bark of *E. ulmoides*. The main parameters such as sample pretreatment, irradiation power, enzyme type, enzyme concentration, pH of enzyme solution, incubation temperature and time, and liquid–solid ratio were systematically studied. The plant samples before and after treatment were observed by scanning electron microscopy (SEM).

## 2. Results and Discussion

### 2.1. Effect of Pretreatment Method

#### 2.1.1. Effect of Irradiation Power

**Figure 1:**
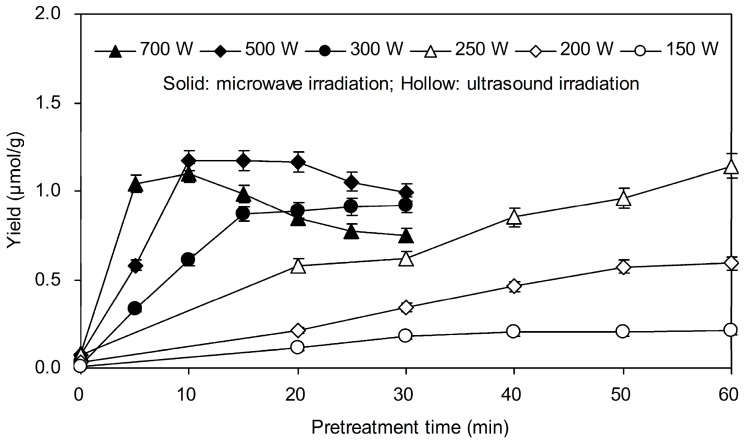
Effect of microwave and ultrasound irradiation power on yield of genipin. One gram degreased sample powder was added into 10 mL deionized water and homogeneous mixing prepared for pretreatment by microwave or ultrasound at different irradiation power. The samples were then mixed with 5.0 mg cellulase, and subsequently the mixtures were incubated at 40 °C for 24 h. After incubation, 10 mL ethanol was added to extract genipin over 30 min.

In this study, in order to improve plant permeability to accelerate enzyme penetration into cell wall, the pretreatment methods including microwave, ultrasound and their combination irradiation were applied and compared. Figure 1 presented the yield of genipin from samples pretreated by microwave and ultrasound irradiation under different irradiation power. For microwave pretreated samples, the yield of genipin obtained at 500 W increased almost linearly from 0.07 ± 0.01 to 1.17 ± 0.03 μmol/g during 0–10 min, and decreased gently when prolonging pretreated time. The yields of genipin obtained under irradiation power 300 W and 700 W were less than that under 500 W irradiation power. A dramatically decrease was observed for 700 W irradiation power treated at 10 min, which is because high energy can lead to the disintegration of genipin. Figure 1 also described the effect of different ultrasound irradiation power on yield of genipin. The yield of genipin increased from 0.07 ± 0.01 to 1.14 ± 0.03 μmol/g when the ultrasound irradiation power increased from 150 to 250 W at 60 min. Therefore, 500 W was selected as the optimal microwave irradiation power, and 250 W was considered as the optimal ultrasound irradiation power.

#### 2.1.2. Effect of Different Pretreated Programs

**Figure 2:**
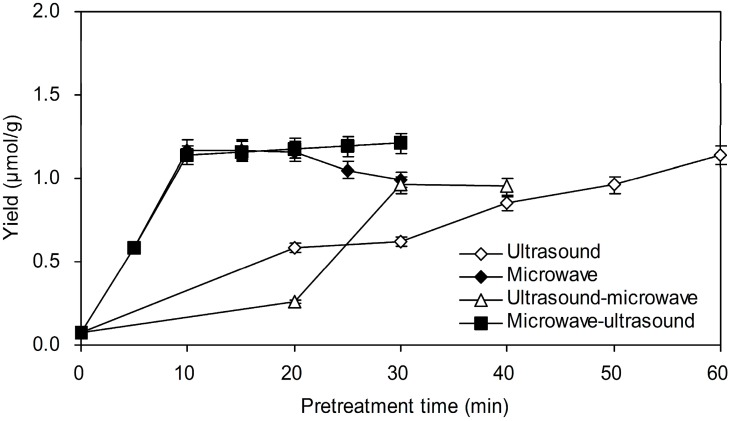
Effect of microwave, ultrasound or their combination irradiation method on yield of genipin. One gram degreased sample powder was added into 10 mL deionized water and homogeneous mixing prepared for pretreatment by microwave, ultrasound or their combination irradiation method. The ultrasound and microwave irradiation power were 250 W and 500 W, respectively. The samples were then mixed with 5.0 mg cellulase, and subsequently the mixtures were incubated at 40 °C for 24 h. After incubation, 10 mL ethanol was added to extract genipin over 30 min.

Results in Figure 2 present the influences of microwave, ultrasound and their combination irradiation methods on yield of genipin. For pretreatment of ultrasound irradiation, the yield of genipin was increased gradually over 60 min. After microwave irradiation, the yield of genipin increased dramatically in the first 10 min, and then decreased from 20 to 30 min. High microwave energy over a long time might lead to the degradation or isomerization of genipin. For the combination irradiation method, the yield of genipin was 1.15 ± 0.03 μmol/g for microwave-ultrasound, which was much higher than 0.58 ± 0.02 μmol/g for ultrasound-microwave. The effect of microwave-ultrasound irradiation on yield of genipin was unapparent compared with that of pretreated by microwave irradiation. The material absorbed microwave energy to facilitate the destruction of the cellular structure and release of active substance out of plant cell. In addition, microwave irradiation could improve enzyme performance, which was detailed in reference [32]. Therefore, microwave irradiation at 500 W for 10 min was selected as the optimal pretreatment method in four alternative offers.

### 2.2. Effect of Enzymatic Hydrolysis Parameters

#### 2.2.1. Selection of Enzyme Type

**Figure 3:**
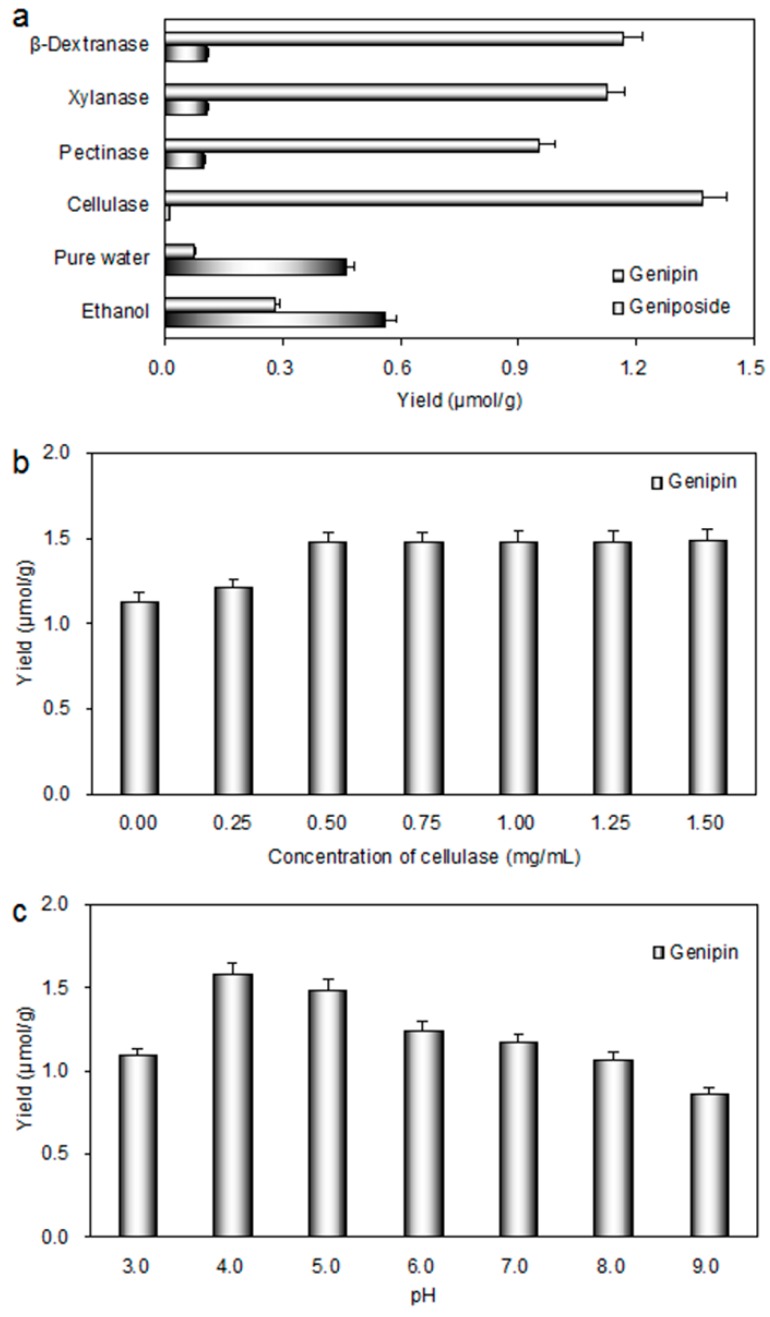
Effect of different parameters in EHSE process on the yield of genipin: (**a**) enzyme types; (**b**) concentration (mg/mL); and (**c**) pH. One gram degreased sample powder was added into 10 mL deionized water and homogeneous mixing prepared for pretreatment by microwave at 500 W for 10 min. Then different enzymes, concentration and pH were set and the samples were incubated at 40 °C for 24 h. After incubation, 10 mL ethanol was added to extract genipin over 30 min.

As is well-known, geniposide can lose a molecule of glucose to produce genipin after hydrolysis by β-glucosidase, which is an equimolar conversion process. In order to compare easily, the “μmol/g” was taken as the unit of extraction yield. As shown in Figure 3a, the extraction yield of genipin extracted in ethanol was much higher than that extracted in water. This illustrated that genipin has better solubility in ethanol. Therefore, after enzymatic treatment, ethanol was added to extract genipin.

It can be seen from Figure 3a that the yield of genipin after incubation with cellulase was 1.18 fold, 1.22 fold and 1.44 fold of those treated with β-dextranase, xylanase and pectinase, respectively. The structural complexity and the rigidity of plant cell wall are major barrier for the release of intracellular constituents [33]. The use of enzyme is helpful to dissolve out intracellular components and to improve the extraction yield [34,35]. Cellulase is a multi-component enzyme system consisting of exoglucanases, endoglucanases, and β-glucosidases. Exoglucanases hydrolyze crystalline cellulose to release cellobiose; endoglucanases preferably attack amorphous cellulose and some short chain oligomers; and β-glucosidases hydrolyse cellobiose into glucose. β-dextranase is an enzyme system that can hydrolyze glucan xylanase systems, including β-1,4-*endo*-xylanase, β-xylosidase, α-l-arabinosidase, and α-d-glucosiduronide as well as is able to degrade hemicellulose. β-1,4-*endo*-xylanase is the most important hydrolase of xylanase systems. It hydrolyzes β-1,4-glycosidic bond of xylan molecules into small oligosaccharides and xylobiose, and a small amount of xylose and arabinose. Pectinase could degrade pectic substances existing in plant tissue. Therefore, cellulase plays two important roles in this article including hydrolyzes the cell wall and the glucosidic bond of geniposide. Hence, cellulase was considered as more efficient for the preparation of genipin.

#### 2.2.2. Effect of Enzyme Concentration

The effect of the concentration of cellulase on the yield of genipin was studied and the results are shown in Figure 3b. It is obvious that the yield of genipin increased gradually with increasing cellulase concentration from 0.00 to 0.50 mg/mL. However, no remarkable improvement for the yield of genipin was observed when the enzyme concentration increased from 0.50 to 1.50 mg/mL. This result indicated that enzyme concentration of 0.50 mg/mL could provide sufficient enzyme activities. Cellulase concentration of 0.25–0.75 mg/mL was selected for the following process.

#### 2.2.3. Effect of pH

Besides enzyme type and enzyme concentration, pH is another important factor. In this study, the optimum pH of cellulase is 4.0–5.5, which is advised by manufacturers. To find out the optimal pH for cellulase, the effect of different pH values on yield of genipin was investigated. Results shown in Figure 3c illustrated that the highest yield of genipin was obtained at pH 4.0. This result can be explained by the fact that pH of the enzyme solution influences the enzyme activity. The effect of pH on enzyme activity was similar to the study by Ticar *et al.* [36]. Therefore, pH 3.0–5.0 was chosen for BBD experiment.

#### 2.2.4. Effect of Incubation Temperature

**Figure 4:**
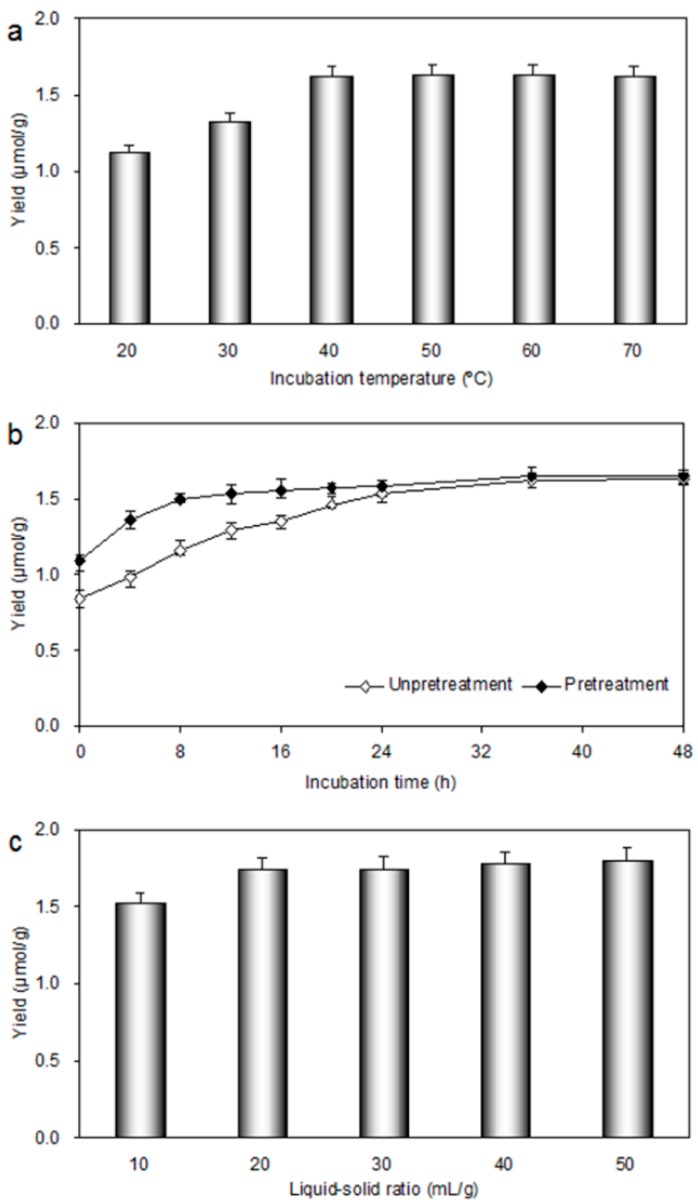
Effect of different parameters in EHSE process on the yield of genipin: (**a**) incubation temperature (°C); (**b**) incubation time (min); and (**c**) liquid–solid ratio (mL/g). Different amounts of degreased sample powder were added into 10 mL deionized water and pretreated with microwave at 500 W for 10 min. The samples were then mixed with 5.0 mg cellulase and incubated at different conditions. After incubation, 10 mL ethanol was added for extracting genipin for 30 min.

Incubation temperature plays an important role in EHSE. The reaction rates of enzymes were accelerated with temperature increas up to an optimum value. Higher or lower temperatures will lead to weak enzyme activity; and too high of a temperature will inactivate the enzyme. As it can be seen from Figure 4a, the yield of genipin was increased gradually with the increase of incubation temperature. The yield of genipin increased dramatically from 1.12 ± 0.03 μmol/g to 1.62 ± 0.05 μmol/g as the temperature increased from 20 °C to 40 °C. When the variable was changed from 40 °C to 60 °C, slight improvements were observed. It is worth noting that the yield of genipin was decreased when the temperature was higher than 60 °C. It was likely that enzymes were heat-sensitive, so high temperatures will inactivate the enzymes. As an appropriate incubation temperature, 30–50 °C was selected and used in the subsequent studies.

#### 2.2.5. Effect of Incubation Time

The results of effect of different incubation time on the yield of genipin are given in Figure 4b. It can be seen obviously that the yield of genipin without pretreatment was 1.63 ± 0.05 μmol/g for 24 h. However, the yield of genipin after pretreatment was 1.50 ± 0.05 μmol/g for only 8 h. Moreover, it was observed that the yield of genipin increased significantly with increase of the incubation time from 0 to 8 h after pretreatment. Longer incubation time, however, did not significantly improve the extraction yield Therefore, 8 h was selected for further experiments.

#### 2.2.6. Effect of Liquid-Solid Ratio

The liquid-solid ratio was a crucial parameter, thus a series of experiments with different liquid-solid ratios were carried out to evaluate its effect on yield of genipin. The results presented in Figure 4c demonstrated that the yield of genipin was increased obviously with the increase of the liquid-solid ratio from 10:1 to 20:1 mL/g, and the highest yield of genipin was obtained when the liquid–solid ratio reached 50:1 mL/g. The yield of genipin was 1.74 ± 0.05 μmol/g when the liquid–solid ratio was 20:1 mL/g, which was 96% of the highest yield. Large solvent volumes could make the operation difficult and lead to unnecessary waste, while small volumes may lead to incomplete extraction Hence, 20:1 mL/g was chosen as optimal liquid-solid ratio.

### 2.3. Optimization Parameters by RSM

To study the interactions between different factors, we optimized the concentration of cellulase (*X*_1_), pH (*X*_2_) and temperature (*X*_3_). From Table 1, the Model *f*-value of 1491.22 implies the model is significant and only a 0.01% chance that a “Model *f*-value” this large could occur due to noise. Values of “Prob > *F*” less than 0.0500 demonstrated model terms are significant. Therefore, *X*_1_, *X*_2_*X*_3_, $X_{1}^{2}$, $X_{2}^{2}$, and $X_{3}^{2}$ are significant model terms. The “Lack of fit *f*-value” of 5.83 indicate there is a 6.07% chance that a “Lack of fit *f*-value” this large could occur due to noise. The “Predicted *R*^2^” of 0.9931 is in reasonable agreement with the “Adjust *R*^2^” of 0.9988. “Adequacy Precision” measures the signal to noise ratio. A ratio greater than 4 is desirable. The ratio of 96.30 indicates an adequate signal. This model can be used to navigate the design space.

**Figure 5:**
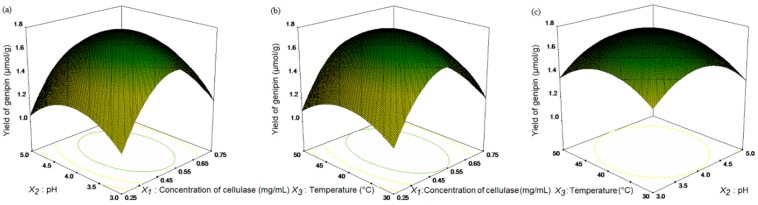
Response surface plot for maximal extraction yields of ginipin as a function of (**a**) enzyme concentration and pH; (**b**) enzyme concentration and temperature; and (**c**) pH and temperature.

The response surfaces for the interaction of independent variables are shown in Figure 5. Figure 5a shows the interaction of concentration of cellulase and pH. Figure 5b presented the interaction of concentration of cellulase and temperature. Figure 5c revealed the interaction of pH and temperature. The yield of genipin can reach to 1.77 μmol/g under the conditions for point prediction by software were: 0.50 mg/mL cellulase concentration, pH 4.0, 40 °C incubation temperature at 20:1 mL/g liquid-solid ratio, 8 h incubation time and 500 W microwave irradiation pretreatment for 10 min.

The verification tests were operated three times under the conditions of point prediction by RSM. The actual yield was 1.71 μmol/g with an error about 0.06 μmol/g.

**Table 1:** Experimental design matrix to screen for variables that determine the yield of genipin and ANOVA results.

Run	BBD Experiments				ANOVA										
	*X*_1_	*X*_2_	*X*_3_	*Y*	Source	Sum of Squares		Degree of Freedom		Mean Square			*f*-Value	*p*-Value	
1	0.75	3.0	40	1.19	Model ^a^	1.24		9		0.14			1491.22	<0.0001	
2	0.50	4.0	40	1.76	*X*_1_	0.028		1		0.028			299.67	<0.0001	
3	0.25	3.0	40	1.07	*X*_2_	0.0003125		1		0.0003125			3.39	0.1081	
4	0.50	4.0	40	1.76	*X*_3_	0.0002		1		0.0002			2.17	0.1842	
5	0.75	5.0	40	1.16	*X*_1_*X*_2_	0.000025		1		0.000025			0.27	0.6185	
6	0.75	4.0	30	1.21	*X*_1_*X*_3_	0.0004		1		0.0004			4.34	0.0757	
7	0.75	4.0	50	1.23	*X*_2_*X*_3_	0.0016		1		0.0016			17.36	0.0042	
8	0.50	5.0	50	1.41	$X_{1}^{2}$	0.79		1		0.79			8577.32	<0.0001	
9	0.25	5.0	40	1.05	$X_{2}^{2}$	0.19		1		0.19			2078.03	<0.0001	
10	0.25	4.0	30	1.11	$X_{3}^{2}$	0.12		1		0.12			1332.28	<0.0001	
11	0.50	5.0	30	1.35	Residual	0.000645		7		0.00009214					
12	0.25	4.0	50	1.09	Lack of fit	0.000625		3		0.000175			5.83	0.0607	
13	0.50	4.0	40	1.76	Pure error	0.00012		4		0.00003					
14	0.50	3.0	50	1.37	Cor total	1.24		16							
15	0.50	3.0	30	1.39	Credibility analysis of the regression equations										
16	0.50	4.0	40	1.77	Index mark	Standard deviation	Mean		Coefficient of variation%		Press	*R*^2^	Adjust *R*^2^	Predicted *R*^2^	Adequacy precision
17	0.50	4.0	40	1.77	*Y*	0.01	1.38		0.70		0.01	0.9995	0.9988	0.9931	96.30

^a^ *X*_1_ is the concentration of cellulase (mg/mL), *X*_2_ is the pH, *X*_3_ is the Temperature (°C), and *Y* is the yield of genipin (μmol/g).

### 2.4. Structural Changes of Samples after EHSE

**Figure 6:**
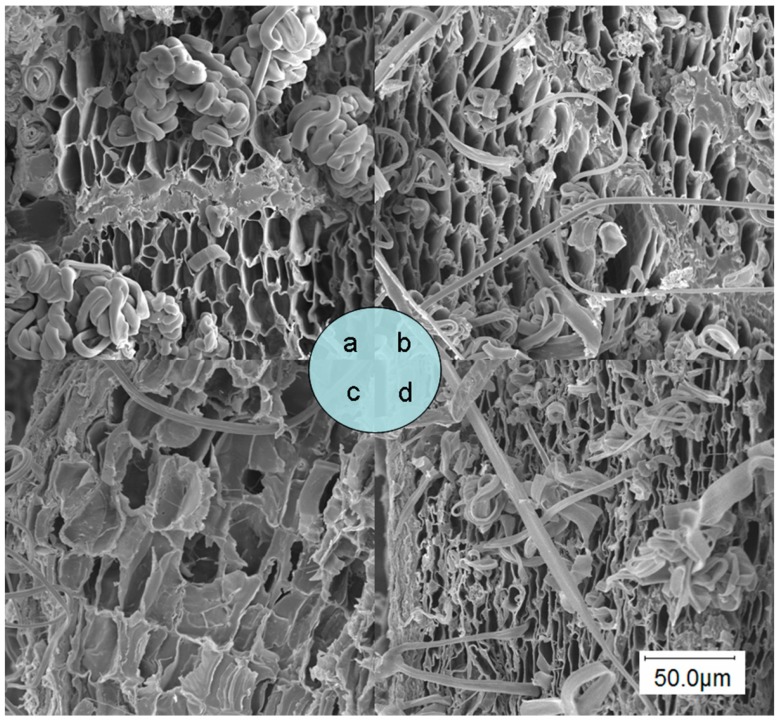
Scanning electron micrographs of bark of *E. ulmoides*: (**a**) raw material; (**b**) degreased sample; (**c**) sample after EHSE; and (**d**) sample after water extraction.

Different extraction procedures may produce distinguishable physical changes in *E. ulmoides* bark. Figure 6 shows scanning electron micrographs of raw material (Figure 6a), degreased sample (Figure 6b), sample after EHSE (Figure 6c) and sample after water extraction (Figure 6d), respectively. Markedly, cell structure of untreated raw material (Figure 6a) was unbroken and a lot of curved filaments were observed. The curved filaments of degreased sample (Figure 6b) were reduced obviously; and the cellular morphology changed little compared with untreated raw material. In the sample after water extraction (Figure 6d), when compared to Figure 6a,b, a partial destruction of the morphological structures of sample was observed. It can be seen from Figure 6c that both the external and internal cells of the sample treated with EHSE were disorganized and damaged significantly. Microwave irradiation lead to the internal thermal of plant material increasing dramatically, and the pressure build-up within the plant material result in expansion, which leads to large molecules of cellulase catalysts permeated easily into cell interior to accelerate the enzymatic degradation process of cytoderm and geniposide. Furthermore, the structure changes of sample surface are in favor of mass transfer of target analytes to the extraction solution.

## 3. Experimental Section

### 3.1. Plant Materials and Chemicals

The bark of *E. ulmoides* was purchased from Sankeshu Medicinal Materials Market, (Harbin, Heilongjiang Province, China) and was ground into a homogeneous size and then sieved (60–80 mesh). Geniposide and genipin reference substances (minimum 98%) were purchased from the National Institute for the Control of Pharmaceutical and Biological Products (Beijing, China). Cellulase (minimum 1000 U/mg), pectinase (minimum 500 U/mg), xylanase (minimum 10,000 U/mg) and β-dextranase (minimum 10000 U/mg) were industrial grade and obtained from Imperial Jade Bio-Technology Co., Ltd. (Yinchuan, Ningxia, China). The advisable individual pH of cellulase, pectinase, xylanase, and β-dextranase were 4.0–5.5, 2.6–6.0, 4.0–7.0 and 6.0–6.5, respectively. Deionized water produced by a Milli-Q purification system from Millipore (Bedford, MA, USA) was used throughout. Methanol and phosphoric acid of chromatographic grade were purchased from J & K Chemical Ltd. (Beijing, China). All the other reagents of analytical grade were obtained from Beijing Chemical Reagents Co. (Beijing, China). All solutions prepared for HPLC analysis were filtered through a 0.45 μm nylon membrane (Guangfu Chemical Reagents Co. Tianjin, China).

### 3.2. HPLC Analysis and Quantification

Chromatographic analyses were performed on a Waters HPLC system consisting of a pump (Model 1525), an auto-sampler (Model 717 plus), and UV detector (Waters 2487 Dual λ absorbance detector, Waters, Sutton, MA, USA). AichromBond-AQ-C_18_ column (5 μm, 4.6 mm × 250 mm, Abel Industries Canada Ltd., Vancouver, BC, Canada) was used for separation of sample solutions.

For HPLC analysis, methanol-water-phosphoric acid (30:69.6:0.4, *v*/*v*/*v*) was used as the mobile phase with 10 µL injection volume. The elution time of each sample was 30 min with 1.0 mL/min flow rate at 25 °C column temperature. The UV detection wavelength was 240 nm for geniposide and genipin. The retention time of geniposide and genipin were 14.9 and 24.4 min, respectively. Standard stock solutions of geniposide and genipin were prepared in methanol and stored at 4 °C. Standard stock solutions were diluted with methanol to different concentration prior to be injected into HPLC. The corresponding calibration curves for each compound were *Y_Geniposide_* = 7964557*X* − 10108 (*R*^2^ = 0.9995) and *Y_Genipin_* = 4484470*X* + 9122 (*R*^2^ = 0.9998). Good linearities were found for geniposide and genipin at the range of 0.02–0.50 mg/mL.

### 3.3. Sample Degreased

The mixture of sample powder (100.0 g) and *n*-hexane (1000 mL) was heating reflux for 2 h. After heating reflux, the mixture was filtered immediately. The above process was repeated twice. The residue was the degreased sample powder that was used throughout.

### 3.4. EHSE for Preparation of Genipin

One gram degreased sample powder was added into 10 mL deionized water and homogeneous mixing. Before enzymatic treatment, each sample was transferred into extraction vessels for pretreatment by microwave, ultrasound or their combination irradiation methods. The digital microwave device (Sineo Chemical Equipment Corp., Shanghai, China) and KQ-250DB ultrasound bath (Kunshan, Jiangsu, China) were used. The ultrasound and microwave irradiation power were 250 W and 500 W, respectively.

The EHSE for preparation of genipin from the bark of *E. ulmoides* was carried out on an HZQ-F160 automatic shaking incubator (Donglian Equipment Technology Corp., Heilongjiang, China). The degreased samples, which were pretreated with optimal pretreatment method, were mixed with 5.0 mg different types of enzyme (cellulase, xylanase, pectinase and β-dextranase). The pH of the mixtures was adjusted by buffered solution; subsequently, the mixtures were incubated under different conditions. After incubation, 10 mL ethanol was added for extracting genipin for 30 min. Water and ethanol were used as control. The solutions were filtered through 0.45 μm nylon membrane prior to HPLC analysis.

### 3.5. Optimization EHSE by Response Surface Methodology (RSM)

Operating conditions of EHSE were optimized by RSM and a Box-Behnken design (BBD) was used for data processing. BBD with three factors is applied using Design-Expert (Version 8.0, Stat-Ease Inc., Minneapolis, MN, USA) without any blocking. The bounds of the factors are 0.25–0.75 mg/mL concentration of cellulase, 3–5 pH, and 30–50 °C temperature.

### 3.6. SEM

Samples with different treatments were scanned by a Hitachi S-520 SEM (Hitachi, San Jose, CA, USA). The samples prepared for SEM were dried at 60 °C for 24 h. They were fixed on aluminum stubs with adhesive tape and then sputtered with gold. Four samples were examined under high vacuum condition at an accelerating voltage of 12.5 kV (50 μm, 500× magnification).

## 4. Conclusions

In this paper, EHSE, which combined microwave irradiation pretreatment, enzymatic hydrolysis and simultaneous extraction into a successive process, was firstly developed for the preparation of genipin from bark of *E. ulmoides*. The optimal conditions for EHSE were evaluated. The genipin yield obtained by EHSE method was 1.71 μmol/g. Scanning electronic microscopy of plant samples showed that the plant material were disintegrated efficiently after EHSE, enhancing the solvent into the plant matrix, thus accelerating the release of intracellular target component. The proposed method of EHSE showed a good alternative technology for the preparation of genipin from bark of *E. ulmoides* as well as other herbs.
